# PTH, FGF-23, Klotho and Vitamin D as regulators of calcium and phosphorus: Genetics, epigenetics and beyond

**DOI:** 10.3389/fendo.2022.992666

**Published:** 2022-09-29

**Authors:** Ignacio Portales-Castillo, Petra Simic

**Affiliations:** ^1^ Department of Medicine, Division of Nephrology, Massachusetts General Hospital and Harvard Medical School, Boston, MA, United States; ^2^ Endocrine Unit, Massachusetts General Hospital and Harvard Medical School, Boston, MA, United States

**Keywords:** calcium, FGF23 (fibroblas growth factor), phosphorus, PTH - parathyroid hormone, Klotho, epigenetic, Vitamin D

## Abstract

The actions of several bone-mineral ion regulators, namely PTH, FGF23, Klotho and 1,25(OH)2 vitamin D (1,25(OH)_2_D), control calcium and phosphate metabolism, and each of these molecules has additional biological effects related to cell signaling, metabolism and ultimately survival. Therefore, these factors are tightly regulated at various levels – genetic, epigenetic, protein secretion and cleavage. We review the main determinants of mineral homeostasis including well-established genetic and post-translational regulators and bring attention to the epigenetic mechanisms that affect the function of PTH, FGF23/Klotho and 1,25(OH)_2_D. Clinically relevant epigenetic mechanisms include methylation of cytosine at CpG-rich islands, histone deacetylation and micro-RNA interference. For example, sporadic pseudohypoparathyroidism type 1B (PHP1B), a disease characterized by resistance to PTH actions due to blunted intracellular cAMP signaling at the PTH/PTHrP receptor, is associated with abnormal methylation at the *GNAS* locus, thereby leading to reduced expression of the stimulatory G protein α-subunit (Gsα). Post-translational regulation is critical for the function of FGF-23 and such modifications include glycosylation and phosphorylation, which regulate the cleavage of FGF-23 and hence the proportion of available FGF-23 that is biologically active. While there is extensive data on how 1,25(OH)_2_D and the vitamin D receptor (VDR) regulate other genes, much more needs to be learned about their regulation. Reduced VDR expression or VDR mutations are the cause of rickets and are thought to contribute to different disorders. Epigenetic changes, such as increased methylation of the VDR resulting in decreased expression are associated with several cancers and infections. Genetic and epigenetic determinants play crucial roles in the function of mineral factors and their disorders lead to different diseases related to bone and beyond.

## Introduction

Complex interplay of the parathyroid hormone (PTH), fibroblast growth factor 23 (FGF23), Klotho and 1,25(OH)2 vitamin D (1,25(OH)_2_D) regulates calcium and phosphate metabolism. However, each of these molecules has additional biological effects beyond bone mineral regulation, related to cell signaling, metabolism and ultimately survival (1). Therefore, these factors seem to be tightly regulated at different levels – genetic, epigenetic, protein secretion and cleavage. In this review, we discuss the main genetic and epigenetic regulatory pathways of PTH, FGF-23, Klotho and 1,25(OH)_2_D, that are clinically relevant for the activity of these hormones. Many of these actions intersect in the proximal renal tubule, where phosphate reabsorption is decreased in response to PTH or FGF-23, and where 1-α hydroxylase is synthesized for the generation of 1,25(OH)_2_D (2). We describe epigenetic mechanisms such as DNA methylation, mRNA stabilization and histone modification that are involved in mineral regulation. Histone modifications include acetylation, methylation, ubiquitylation, phosphorylation, all of which might affect accessibility to DNA. For example, histone deacetylases (HDAC) promote DNA condensation, suppressing DNA transcription (3).

We also briefly discuss endochondral bone formation, as it relates to the actions of the parathyroid hormone receptor (PTH1R), a critical G-protein coupled receptor that mediates the function of PTH and the parathyroid hormone related peptide (PTHrP). The physiologic mechanisms downstream of mineral hormones that govern calcium and phosphorus homeostasis will only be briefly discussed.

## The parathyroid hormone synthesis and secretion

The parathyroid hormone (PTH) is critical to maintain normal levels of serum calcium. In case of parathyroidectomy, and limited external calcium supply, death ensues within hours from severe hypocalcemia and hyperphosphatemia, unless treated with PTH (4). Therefore, it’s not surprising that PTH secretion appears to be a highly regulated process. The PTH gene in humans consists of three exons that span about 4 kb on chromosome 11p15, with exon 2 encoding the majority of the prepropeptide sequence and exon 3 encoding the amino acids of the mature peptide (5). Transcription of the PTH gene is stimulated mainly by hypocalcemia (6, 7), but also by hyperphosphatemia (8), uremia (9–11), and is suppressed by 1,25(OH)_2_D (6). While patients with advanced renal disease often have elevated levels of PTH, the relevance of a possible stimulatory effect of uremia on PTH synthesis remains undefined, as patients with renal disease have other abnormalities that might contribute to PTH elevation. When serum calcium increases, it binds to the extracellular domain of Ca^2+^-sensing receptor (CaSR) activating intracellular pathways that result in a decrease in PTH secretion, whereas a decrease in Ca^2+^ releases this suppression, to promote tonic PTH secretion (12). The PTH response to calcium is highly dependent on the CaSR, as demonstrated by diseases associated with gain or loss of function mutations in CaSR, which cause hypocalcemic or hypercalcemic disorders, respectively (12, 13). Interestingly, it has been recently shown that phosphate might have an inhibitory effect on the CaSR independent of calcium, thereby providing a mechanism for PTH secretion in response to hyperphosphatemia (14). After transcription of the PTH gene, mRNA is partly degraded by cytosolic proteins that bind to the 3’-untranslated region (UTR) (15). Among such cytosolic proteins the AU-binding factor 1 (AUF1) and N-ras are PTH mRNA-stabilizing proteins, while the K-homology splicing regulatory protein (KSRP) is a destabilizing protein (15). Conditions such as hypocalcemia or uremia can increase the amount of PTH mRNA by modifying the activity of mRNA binding proteins (16, 17).

The PTH protein is synthesized as a 115 amino acids preprohormone. The 25-amino acid signal pre-sequence is cleaved in the endoplasmic reticulum (ER), and subsequently residues -6 through -1 of the prohormone (PTH -6-84) are cleaved in the Golgi apparatus by proprotein convertases, among them being furin the most efficient (18). The mature, active, hormone circulates as an 84 amino acid protein (PTH 1-84) (19). A few mutations have been described in patients, associated with abnormal processing of prepro-PTH (5), or affecting the mature PTH protein, resulting in hypoparathyroidism (20). For example, amino acid substitution from cysteine to arginine in the preprohormone, disrupts the hydrophobic core of the signal sequence and such mutation leads to inefficient protein processing in the ER (21). Other mutations affecting residue 1 (near the site of cleavage of pro-PTH) or residue 56 of the mature version of the hormone have been found in patients with idiopathic hypoparathyroidism (IPH) (5, 22). Interestingly, in patients with the mutations at residue 1 or residue 56, the different available PTH immunoassays show very variable levels of serum PTH, going from below normal levels (consistent with hypoparathyroidism) to elevated levels (as in pseudohypoparathyroidism) depending on the affinity and target site of the antibody used to detect the circulating PTH (5, 20) **Table 1**. Therefore, these diseases illustrate the importance of normal processing of the PTH preprohormone and the potential limitations of current assays to accurately quantify the biologically active portion (PTH 1-84) of circulating PTH polypeptides.

**Table 1:** Clinical examples of genetic and epigenetic disorders involving PTH, FGF-23 and Vitamin D.

PTH and PTHrP signaling disorders			Pathogenesis
**GENETIC**	Idiopathic hypoparathyroidism due to PTH mutations		Mutations in preproPTH lead to inadequate processing to the mature hormone in the endoplasmic reticulum. Only one mutation described in the mature PTH, (Cys25]PTH.
**EPIGENETIC**	Pseudohypoparathyroidism type 1B due to maternal deletions that affect *GNAS* methylation (i.e STX16 deletions)		Abnormal methylation in the *GNAS* locus from the maternal allele suppresses Gsα expression leading to PTH resistance (paternal allele is normally downregulated in proximal renal tubule).
**FGF-23 and Klotho signaling**			
**GENETIC**	Hyperphosphatemic familial tumoral calcinosis		Reduced intact FGF-23 as seen in mutations in FGF-23 or GALNT3, which results in increased cleavage of FGF-23. Loss of function mutations in Klotho impair FGF-23 action in the proximal renal tubule.
	Genetic causes of hypophosphatemia with high FGF-23 levels		A cleavage-resistant FGF-23 mutant in ADHR is associated with hypophosphatemia Several other conditions (i.e XLH, ARHR1, ARHR2) increase FGF-23 by less defined mechanisms
**EPIGENETIC**	Decreased klotho levels in CKD		Uremic toxins increase methylation and histone deacetylation of klotho gene, reducing expression of klotho and contributing to resistance to FGF-23 actions in the proximal tubule
**Vitamin D signaling**			
**GENETIC**	Hereditary vitamin D-resistant rickets	VDR gene mutation leading to hypocalcemia, secondary hyperparathyroidism, and severe early age rickets.	
**EPIGENETIC**	Methylation changes in acquired conditions	Increased methylation of VDR is associated with cancer and infections	

PTH, parathyroid hormone; Gsα, alpha subunit of stimulatory G protein; FGF-23, Fibroblast growth factor 23; GALNT3, uridine diphosphate-N-acetyl-alpha-D-galactosamine:polypeptide N-acetylgalactosaminyltransferase 3; ADHR, Autosomal dominant hypophosphatemic rickets; XLH, X-Linked Hypophosphatemia; ARHR1, Autosomal recessive hypophosphatemic rickets type 1; ARHR2, Autosomal recessive hypophosphatemic rickets type 2; VDR, vitamin D receptor.

After intracellular processing of the prohormone, the mature polypeptide (84 amino acids) is packaged in secretory granules in the cytosol. Intracellularly, PTH(1-84) is further cleaved by proteases, releasing biologically inactive C-terminal portion to the circulation, which serve as an additional regulatory step (23). Additional post-translational modifications of the circulating PTH peptide have been demonstrated including phosphorylation and oxidation, which might affect biologic activity or detection by current PTH assays (24, 25).

## Normal methylation at the *GNAS* locus is required for PTH1R signaling in the kidney

**Figure 1:**
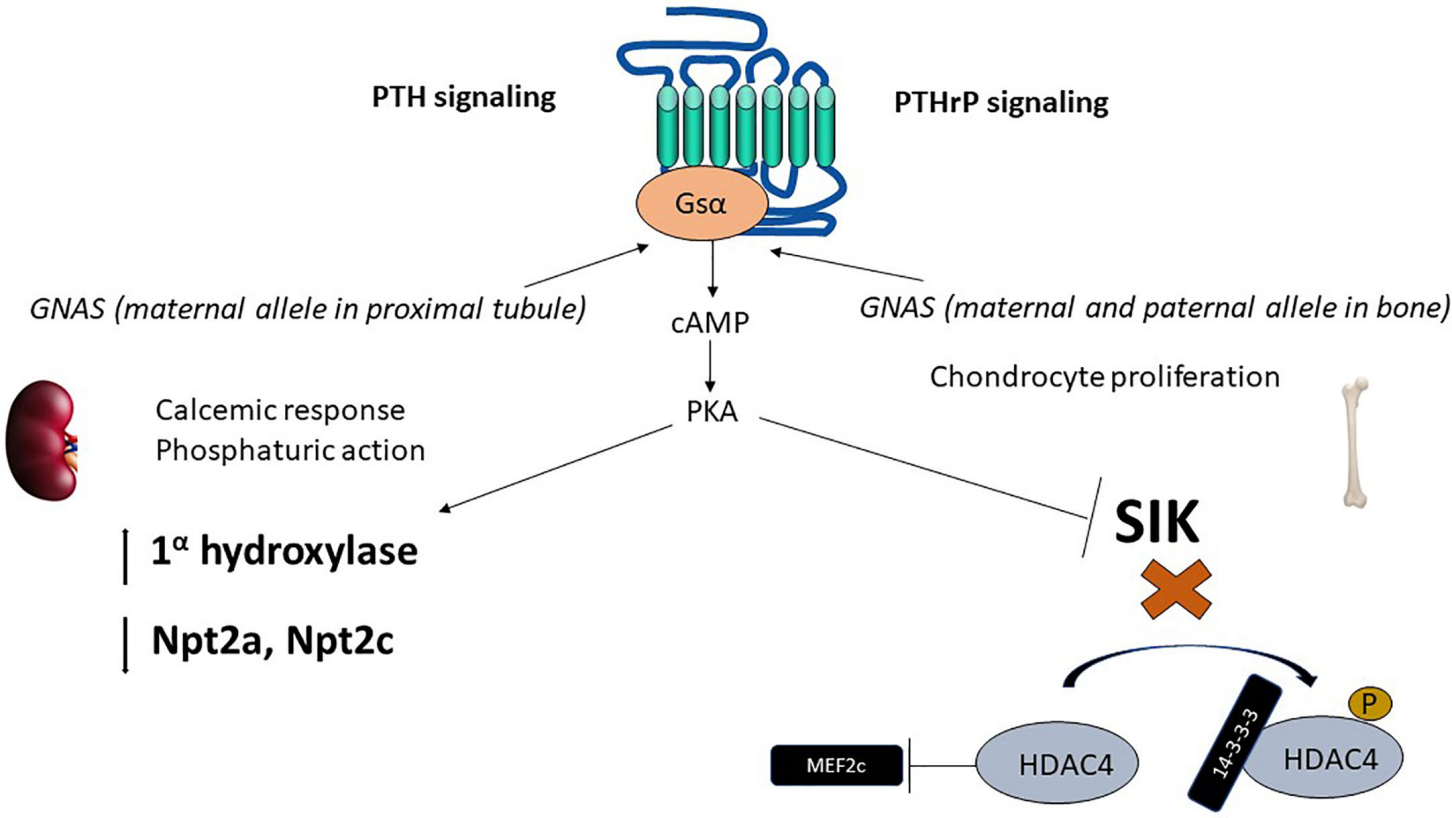
Epigenetics of PTH and PTHrP signaling. The parathyroid hormone receptor (PTH1R) mediates the actions of two ligands, PTH and PTHrP, with independent roles in calcium homeostasis and bone developments, respectively. PTH1R main signaling pathway in response to PTH or PTHrP requires activation of the α subunit of the heterotrimeric stimulatory G-protein (Gsα). Gsα is encoded by GNAS, a complex locus that normally undergoes methylation on the maternal side (for normal expression of Gsα). In the proximal tubule, Gsα is almost exclusively derived from the maternal side during adult life. cAMP generation results in phosphaturic action (via downregulation of phosphate transporters Npta and Npt2c) and vitamin D activation. In the developing bone, downstream of Gsα, cAMP production activates PKA and inhibits SIK thereby exerting a control in MEF2c, which results in chondrocyte proliferation. *PTH, parathyroid hormone; PTHrP, parathyroid hormone related peptide; PKA, protein kinase A; SIK, salt inducible kinase; HDAC, Histone deacetylase; MEF2, myocyte enhancer factor.*.

The parathyroid hormone receptor (PTH1R) is a member of the class B or secretin family of G-protein coupled receptors (GPCR) (26) that mediates the actions of PTH as well as the parathyroid hormone related peptide (PTHrP) (19). While PTH main actions are related to maintain normal calcium levels, PTHrP principal biologic function is to regulate endochondral bone formation (27). The main intracellular signaling pathway activated by either peptide when bound to PTH1R, namely cAMP production, requires activation of the stimulatory subunit of the G-protein (Gsα) **Figure 1**.

Patients with pseudohypoparathyroidism (PHP) have hypocalcemia and hyperphosphatemia, similar to those of hypoparathyroidism, but have high levels of biologically active PTH (28). Most identified cases of pseudohypoparathyroidism type1a (PHP1A) are due to loss-of-function mutations in Gsα (28, 29). Gsα is encoded by the *GNAS* locus, a complex locus on chromosome 20q that generates 3 additional transcripts (A/B, extra-large form of Gsα (XLαs) and neuroendocrine secretory protein 55 (NESP55). Loss-of-function mutations in the *GNAS*, when inherited from a female, result in PHP with skeletal abnormalities that are consistent with Fuller Albright’s description of PHP, and hence these constellations of clinical findings are currently known as Albright’s hereditary osteodystrophy (AHO) or PHP1A (30). Interestingly, if the *GNAS* mutation is instead derived from the male, there is no resistance to PTH actions in the kidney, but only the skeletal abnormalities, which is known as pseudopseudo-hypoparathyroidism (PPHP).

Most cases of pseudohypoparathyroidism type 1B (PHP1B) are sporadic, whereas autosomal dominant PHP is less common and often associated with maternal derived mutations in *STX16.* Both forms of PHP1B are associated with abnormal methylation (both abnormal gain and loss of methylation) at differentially methylated regions (DMR) in *GNAS* exons. Such methylation changes, by unknown mechanisms, suppress Gsα expression from the maternal side. Given that the paternal allele of Gsα is normally progressively suppressed with age in the proximal renal tubule, suppression of Gsα derived from the mother results in deficient Gsα expression in the proximal renal tubule, and thus impaired response to PTH in patients with PHP1B (31, 32).

## The role of PTHrP, PTH1R and histone deacetylases in bone development

PTHrP acting on PTH1R is necessary to maintain chondrocyte proliferation during bone development (33) **Figure 1**. Mice with knockout of the PTH1R or PTHrP die *in utero* or shortly after birth, and share a common phenotype characterized by accelerated mineralization of bones formed by endochondral replacement (27). In humans, homozygous mutations in the PTH1R gene resulting in severe loss of function are lethal, as seen in Blomstrand Chondrodysplasia (BLC) (34). Heterozygous mutations in PTH1R are compatible with life and associated in humans with primary failure of tooth eruption (35, 36).

Similarly to PTH, PTHrP also predominantly signals *via* cAMP (26). The downstream effects of increased cAMP are protein kinase A (PKA) activation and inhibition of salt-inducible kinases (SIK) (37). The inhibitory effect on SIK, upon PTH1R activation, favors dephosphorylation of the class II histone deacetylases HDAC4 and HDAC5 (38). HDAC4 and HDAC5 are class II histone deacetylases, that unlike class I histone deacetylases, have only modest deacetylase function (gene suppression). Instead, class II histone deacetylases have N-terminal extensions that bind 14-3-3 proteins in the phosphorylated state (39). Upon dephosphorylation of HDAC4/HDAC5 due to SIK inhibition, the 14-3-3 proteins are released, and the free N-terminal extension of HDAC4/HDAC5 bind and inactivate transcription factors, such as myocyte enhancer factor 2 (Mef2), which exerts a control on chondrocyte hypertrophy (39). *HDAC4* knockout mice have accelerated chondrocyte hypertrophy and die prematurely with a similar phenotype as PTHrP or PTH1R knockout animals, consistent with PTHrP, PTH1R and HDAC4 sharing a common signaling pathway (38).

## FGF-23 regulates serum phosphate and increases during renal injury

FGF23 is primarily a bone- and bone marrow-derived hormone, with 251 amino acids, which is critical to maintain phosphate homeostasis. FGF23 decreases phosphate reabsorption and 1,25(OH)_2_D synthesis in renal proximal tubules (40). FGF-23 was first identified in families with autosomal dominant hypophosphatemic rickets (ADHR) (41), associated with missense mutations in FGF-23 at positions 176 or 179 (R176Q/W and R179Q/W) that render this peptide cleavage resistant and thus increase the intact portion of FGF-23 (iFGF-23), which is the biologically active peptide and thus result in hypophosphatemia (41). Using site-specific antibodies that bind either to the N-terminal or C-terminal site of FGF-23, it was found that FGF-23 is cleaved intracellularly mainly by the pro-protein convertase furin at the consensus site, but additional proteases including tissue-type PA (tPA) and urokinase-type PA (uPA) have now also demonstrated to cleave (inactivate) FGF-23 under experimental conditions (42–44).

Phosphate consumption increases dose-dependently mRNA abundance of FGF-23 in the bone as well as circulating iFGF-23 (2, 45). The increase in iFGF-23 in response to phosphate levels is at least in part dependent on the bone Na+-Pi co-transporter PiT2/Slc20a2 and the FGF receptor (FGFR1c). For example, global knock out of PiT2 in mice is associated with inappropriately normal levels of FGF-23 when these mice are fed with a low phosphate diets (46). Intracellular phosphate has ligand-independent effects on FGFR1c by receptor phosphorylation, which activates the ERK pathway and the transcriptional activators EGR1 and ETV5 resulting in increased expression of polypeptide N-acetylgalactosaminyltransferase 3 (GALNT3), which catalyzes the protective O-glycosylation in FGF-23, preventing cleavage by furin (47). Thus, acting on two different surface receptors, phosphate enhances transcription and prevents cleavage of FGF-23 (48). Additional regulation of cleavage is provided by phosphorylation of serine 180 by Fam20C, which prevents O-glycosylation, hence favoring cleavage (48), presumably when serum phosphate is low.

**Figure 2:**
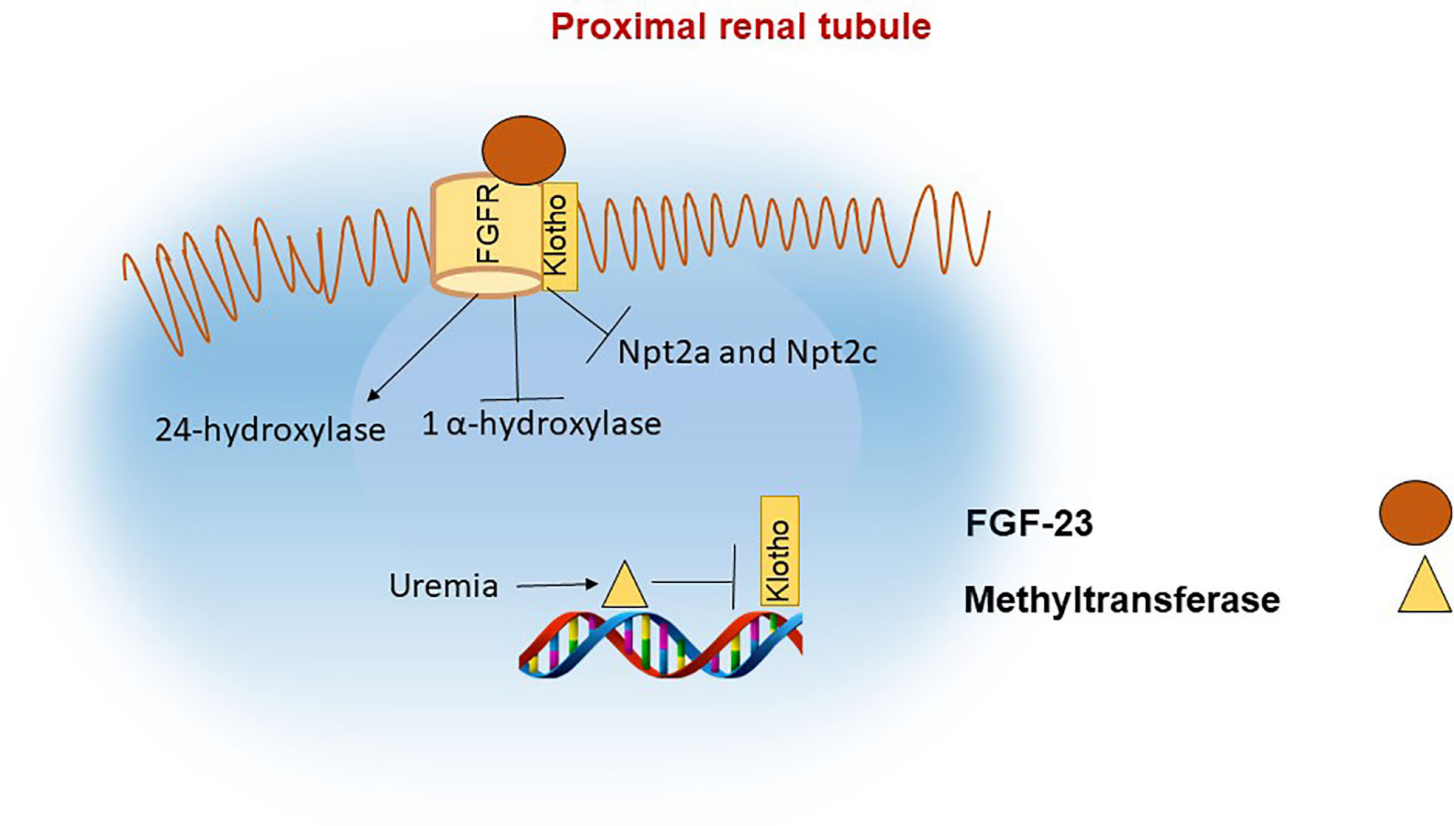
FGF-23 actions in the proximal renal tubule. In the proximal renal tubule FGF-23 binds to the FGF receptor and its coreceptor Klotho. The actions of FGF-23 include inhibition of 1α hydroxylase and of the phosphate channels NPT2a and NPT2c with the net effect of decreasing phosphate reabsorption in the kidneys and indirectly reducing phosphate absorption in the intestine due to reduced levels of 1,25(OH)_2_D. Klotho synthesis is reduced in renal failure, in part as a result of increased methylation of the klotho gene.

In the kidney, FGF-23 binds to the FGF receptor (FGFR) and its co-receptor klotho (49, 50). The intracellular actions of FGF-23 decrease the membrane availability of the phosphate channels in the brush border membrane (BBM) of the proximal renal tubule, namely the type II sodium-phosphate co-transporters Npt2a and Npt2c (51). FGF-23 also downregulates the activity of 1-apha hydroxylase and upregulates 24-hydroxylase, thereby decreasing the levels of 1,25(OH)_2_D (2, 52) **Figure 2**. The decrease in functional vitamin D limits the absorption of phosphate and calcium in the intestine (53).

Both acute and chronic kidney injury increase circulating FGF23 levels as mechanism to prevent hyperphosphatemia (54). However, high FGF-23 levels correlate with morbidity and mortality in patients with renal disease (54–57). The mechanisms how kidney injury regulates FGF23 remain largely unknown. We performed a comprehensive metabolomic analysis in individuals undergoing renal arterial and vein blood sampling, to identify renal derived metabolites that correlate with circulating FGF-23. This led to the identification of glycerol-3-phosphate (G3P), a kidney derived metabolite, that increases during acute kidney injury (AKI) in human subjects and that parallels the increase in serum FGF-23 (58). When injected to animals, G3P transitions to lysophosphatidic acid in the bone and bone marrow, where it is required for VDR induced FGF23 transcription at -395 to -311 promoter site (58).

In order to identify additional transcription enhancers of FGF-23, which control the response to phosphorus levels or that are active in patients with chronic kidney disease (CKD), Onal et al. used chromatin immunoprecipitation with the antibodies CTCF, H3K9ac, H4K5ac, H3K4me1, H3K4me2, and H3K27ac, followed by DNA sequencing (ChIP-seq). This technique led to the identification of a gene region 16 kB upstream from FGF-23, as a putative enhancer (59). Consistent with its regulatory role, global knock-out of this region prevents the early increase in FGF-23 transcription in mice with CKD. Three additional epigenetically marked regions were tested for the contribution to FGF-23 secretion in response to phosphate or 1,25(OH)_2_D. Among these, the deletion of an enhancer region in close proximity to FGF-23, almost completely blunted the transcription increase of FGF-23 in the bone in response to a high phosphate diet or 1,25(OH)_2_D injection (60).

## Additional control of FGF-23 secretion, beyond phosphate and renal disease

*In vivo*, other positive regulators of FGF-23 include inflammatory related cytokines such as IL-1β, TNF-α (61), lipopolysaccharides (LPS) (62), mineral mediators like parathyroid hormone (PTH) (63), 1,25(OH)_2_D and hematopoietic factors such as hypoxia or iron deficiency (64). The effect of inflammation or iron deficiency is mediated by an increase in hypoxia-inducible factor α (HIF1α) abundance or stabilization (65, 66). Several investigations have demonstrated the role of HIF1α in FGF-23 stimulation. In osteoblasts cell lines, HIF1α increases the FGF-23 promoter activity and results in increased FGF-23 transcription and secretion, which is completely reversible with HIF1α blocking inhibitors (66). In contrast to phosphate mediated stimulation of FGF-23, HIF1α also promotes the cleavage of iFGF-23, such that the increase in the biologically active portion of FGF-23 is attenuated (62). Interestingly, in patients with ADHR, urine phosphate wasting exacerbates during periods of iron deficiency. This is because iron deficiency stimulates HIF1α and cleavage is prevented in this mutant form of FGF-23, thus increasing the amount of iFGF-23 (67).

In contrast to iron deficiency, which typically only elevates C-terminal FGF-23, some iron preparations have been commonly associated with hypophosphatemia *via* an increase in iFGF-23. For example, in a pooled analysis of clinical trials, 41% of patients treated with ferric carboxymaltose developed hyperphosphatemia predominantly within 2 weeks of treatment (68, 69).

Supporting the notion that there are multiple pathways involved in FGF-23 upregulation beyond phosphate sensing or HIF1α, conditional deletion of HIFα (HIF1α/Osteocalcin (OCN)-Cre) does not reduce FGF-23 levels in the *Hyp* mice (64), an animal model of X-linked hypophosphatemia (XLH). XLH in humans is caused by deletion of the phosphate-regulating endopeptidase homolog X-linked (PHEX) and is associated with high levels of FGF-23. The physiologic function of PHEX and how its loss results in increased FGF-23 is not well understood. PHEX has been shown to cleave proteins involved in bone remodeling and mineral balance such as osteopontin and the parathyroid hormone related peptide (PTHrP (70)) but has not been consistently found to have an important role in FGF-23 cleavage (71, 72).

## Klotho is modified at the epigenetic level in renal disease

Klotho is the FGF-23 coreceptor and a secreted protein, which was incidentally discovered in mice with features of premature aging. There are 3 isoforms of this protein (alpha, beta and gamma) (73). While klotho is mainly expressed in the distal tubule, the FGF-23 actions on phosphate transporters and vitamin D metabolism occur in the proximal tubule. Ablation of klotho in the proximal tubule of mice results in hyperphosphatemia upon challenge with a high phosphate diet only, consistent with a biologically relevant role of klotho in the proximal tubule (74).

Lack of klotho in humans or mice leads to severe hyperphosphatemia, increased 1,25(OH)_2_D and calcium levels, similar to FGF-23 deficiency. During CKD progression, α-klotho levels decline in parallel with increases in FGF-23 (75). Klotho expression in CKD appears to be regulated through epigenetic mechanisms (76) **Figure 2**. For example, mice exposed to uremic toxins have increased levels of DNA methyltransferase (DNMT1) which leads to DNA hypermethylation of the klotho gene and consequently lower abundance of klotho protein (77, 78).

In mice with folic acid induced AKI, the activity of the histone deacetylases HDAC1 and HDAC1 was shown to be elevated and correlated with a decrease in Klotho expression. Thus, histone deacetylation decreases the accessibility of transcription factors to DNA, thereby suppressing expression (79).

Similar to the original descriptions of klotho leading to premature senescence and hyperphosphatemia, patients with CKD suffer from a shorter life span, vascular calcifications, and bone disorders (80). The contribution of klotho deficiency to these manifestations have been supported by clinical observations and animal models. For example, pharmacologic targeting of deacetylation and methylation increased klotho levels and reduced renal fibrosis in mice with unilateral ureteral occlusion (81).

## Vitamin D regulation

Vitamin D is generated from skin in response to sun UV light in the form of cholecalciferol (D3) and from diet *via* intestinal absorption in the forms of ergocalciferol (D2) from plants and D3 from animals (82). Vitamin D then circulates to liver to get activated to 25-hydroxyvitamin D and subsequently to kidney proximal tubules to become active 1,25(OH)_2_D or calcitriol (83). 1,25(OH)_2_D increases reabsorption of calcium and phosphate in the intestine, calcium reabsorption in the renal distal tubules and has crucial role in growth and development of bones and teeth. The majority of vitamin D comes from UVB photosynthesis (90-100%) (84) and it is regulated by skin color and latitude. The high melanin content of darker skin types blocks UVB, producing less vitamin D, and the lower melanin content of lighter skin allows for more UVB penetration, producing more 1,25(OH)_2_D (85). Skin pigmentation serves as evolutionary mechanism to keep optimal vitamin D levels in the body. 1,25(OH)_2_D deficiency in early childhood can lead to rickets, while in adults leads to osteoporosis and osteopenia.

At the cellular level 1,25(OH)_2_D binds to vitamin D receptor (VDR) and regulates VDR expression (86). VDR is a nuclear receptor and transcriptional regulator that regulates expression of more than 900 genes. In addition, VDR has calcitriol independent effects, as shown in the model of alopecia that cannot be rescued by calcitriol (87). VDR in the complex with retinoid X receptor acts as a ubiquitous transcription factor (83). It activates or represses numerous target genes by binding to vitamin D responsive elements (VDREs) in their promoters (88, 89). By this mechanism, VDR regulates the expression of genes involved in essential biological processes, including calcium and phosphate metabolism, cell cycle, organ development and immunity (83). Therefore, 1,25(OH)_2_D deficiency is implicated in cancer development, immunity and infectious diseases (83).

Genetic mutations in VDR with loss of function cause hereditary vitamin D-resistant rickets (HVDRR) (90). HVDRR is characterized by hypocalcemia, secondary hyperparathyroidism, and severe early-onset rickets. Affected children may also exhibit alopecia. Patients with HVDRR are resistant to 1,25(OH)_2_D treatment and require high dose calcium supplementation.

## Epigenetic regulation of VDR in acquired diseases

VDR gene expression is regulated by four promoters giving rise to 12–14 alternatively spliced transcripts in a tissue specific manner (91). VDR promotor activity is regulated at the epigenetic level by methylation, acetylation, phosphorylation and sumoylation.

## Methylation

DNA methylation of promotor regions usually leads to reduction of gene expression. Changes in methylation of VDR were found in many diseases, like cancer, infectious diseases, immune disease, multiple sclerosis, and kidney stones.

### Cancer

Methylation of VDR has been shown in different cancer types. Patients with adrenocortical carcinoma were found to have higher methylation of cytosine nucleotide of CpG islands in VDR promoter in adrenal glands, leading to reduction of VDR protein and loss of its protective role against malignant growth (92). Similarly, pediatric adrenocortical tumors with high VDR promoter methylation had lower VDR mRNA levels and correlated with advanced disease and reduced survival in these patients (93). Conversely, hypomethylation of VDR promoter in adrenocortical adenoma tissue correlated with more differentiation and aldosterone production from those tumors (94). Methylation of VDR promoter was shown to be important in acute myeloid leukemia cells and DNA methyltransferase inhibitor 5-aza induced VDR expression (95). Use of VDR agonists with hypomethylating agents decreased tumor burden in acute myeloid leukemia mouse models (95).

There is a lot of data about VDR methylation in other cancers as well and most of it is at the observational level. For example, epigenetic profiling of primary melanoma identified VDR hypermethylation important for melanoma progression and was associated with worse survival (96) in a VDR-dependent manner (97). Methylation specific PCR of colorectal cancer tissue vs surrounding healthy tissue showed that hypermethylation of VDR inversely correlates with VDR expression and it is associated with tumor staging (98). Decreased methylation status of VDR in colorectal cancer tissue correlated with longer overall survival (98). In patients with hepatocellular carcinoma the percentage of VDR gene promoter methylation was significantly higher than in the control group of patients (99).

### Infectious diseases

VDR is known to be implicated in several infectious diseases, like tuberculosis, HIV, COVID-19, EBV etc. Methylation of VDR has been shown to play the role in some of those conditions, like tuberculosis and HIV. In children with active tuberculosis, there was more VDR DNA methylation, that was associated with reduced VDR expression and could be associated with increased susceptibility to tuberculosis (100). On the other hand, VDR promoter was hypomethylated in children with EV71-associated severe hand, foot and mouth disease as compared to healthy controls (101).

HIV induced hypermethylation of VDR in T cells led to reduction of VDR, which could mediate T cell apoptosis (102). HIV infected podocytes were shown to have increased expression of DNA methyltransferase and accordingly increased CpG methylation at VDR promoter, repressing VDR expression (103).

### Immune disease

There are some examples of the role of VDR methylation in immune mediated diseases. For example, cumulative methylation level of all CpG sites in VDR promoter was significantly reduced in patients with rheumatoid arthritis vs control patients (104). VDR promoter at exon 1c showed increased DNA methylation levels in T cells from patients with multiple sclerosis compared to controls, with 6.5-fold increase in VDR mRNA levels (105).

### Miscellaneous

Promoter hypermethylation of two target regions in VRD has been shown to be increased in patients with recurrent kidney stones formations vs controls (106).

## Acetylation

Acetylation at lysin residues is an important mechanism of transcriptional factor regulation. There are several lysine acetylation sites identified in VDR. In HEK293 cells, VDR is acetylated at lysine 413 (K413) and is deacetylated by sirtuin-1 (SIRT1) deacetylase (107). SIRT1 overexpression in these cells led to VDR deacetylation and increased transcription. Non-acetylable VDR mutant (K413R) had enhanced responsiveness to 1,25D, suggesting that acetylation of VDR modulates the response to 1,25D (107). On the other hand, resveratrol, SIRT1 deacetylase stimulator, potentiated VDR signaling (108).

VDR has an important role in inflammatory response of beta-cells in diabetes type 2. Acetylation of lysin 91 (K91Ac) in VDR serves as a docking site for one of ATP-dependent chromatin remodeling complexes, BAF complex, important in diabetes (109). Mutation of K91 to alanine (K91A) or arginine (K91R) in Vdr gene significantly reduced the interaction with BAF complex, as well as the total acetylation level of VDR. Binding of BAF complex attenuated VDR activity, while inhibition of VDR-BAF improved beta cell survival and activity, improving glucose levels in db/db diabetic mouse model (109).

## The effect of histone modification on response to VDR

1,25(OH)_2_D3 modulates histone marks of active chromatin at promoter and enhancer regions. The epigenome of human monocytes revealed 550 histone markers of active promoter regions (H3K4me3) and 2473 histone markers of active enhancer regions (H3K27ac) responsive to 1,25(OH)_2_D3 (110). Further, colocalization of VDR and transcription start site of identified regions highlighted 260 and 287 regions with H3K4me3 and H3K27ac modifications, respectively, that were identified on 59 promotors or enhancers of VDR responsive genes. This is the way how histone modification epigenetically modulates the effect of VDR (110).

Histone acetylation usually leads to activation of transcription (111) and is regulated by interaction between histone acetyltransferases and deacetylases. 1,25(OH)_2_D3 directly affects some VDR coactivators with acyltransferase activity, like H3K27ac at the promoter of several VDR target genes (112). Class I histone deacetylase inhibitor, MS-275, reduced colitis activity in a mouse model of ulcerative colitis. The effect of histone H3 deacetylase was blocked in *Vdr-/-* mice, suggesting that histone deacetylation by MS-275 alleviated colitis by activating VDR (113). Histone methylation leads to both gene activation or repression, depending on the histone site that is methylated and it is regulated by methyltransferases and demethylases (114). For example, 1,25(OH)_2_D3 induced the expression of the histone demethylase KDM6B that demethylates H3K27me3, a histone mark that correlates with gene repression. Overall, modification of histone by chromatin regulators affects VDR governed gene expression.

## Phosphorylation

There are several phosphorylation sites in VDR protein, which are responsive to different kinases. PKC-β was shown to phosphorylate serine at position 51 in VDR (115). Phosphorylation resistant mutation of serine to glycine in this region led to decreased *Vdr* transcription in response to calcitriol. Therefore, phosphorylation of serine 51 by PKC-β could play a role in diseases requiring *Vdr* transcriptional activation. PKA phosphorylates serine at position 182 and decreases heterodimerization with RXR, decreasing transactivation by calcitriol (116). However, opposite result was found in rats, where PKA was shown to upregulate *Vdr* transcription in response to PTH (117). Casein kinase II phosphorylates serine at position 208 (118). Replacement of serine with glycine at this position led to decreased *Vdr* transcriptional activity in response to calcitriol. Subsequently, phosphatase inhibitor, okadaic acid, was shown to increase VDR response to calcitriol (119). ATM (ataxia telangiectasia mutated) kinase (DNA-damage response kinase) was shown to phosphorylate serine 208 and 222 of VDR, which impairs the effect of ATM on VDR transactivation activity (120). Calcitriol induces ATM in a positive feedback loop, which might suggest positive role of VDR in carcinogenesis.

## Sumoylation

Sumoylation is a process of binding Small Ubiquitin-like modifier (SUMO) to lysine in transcription factors, which modifies their activity. It was shown that protein inhibitor of activated STAT 4 (PIAS4) sumoylates VDR with SUMO 2 and inhibits is transcription (121). The same group subsequently identified sentrin/SUMO specific protease 1 and 2 (SENP1 and SENP2) to reverse SUMO2 binding to VDR (122). They identified lysine 91 as a likely VDR site that gets sumoylated. It is not certain whether VDR can be glycosylated. There is one *in vitro* study that showed OGlcNAcylation of VDR in THP1 cells and in human macrophages, without correlation to downstream signaling or physiologic conditions (123). In conclusion, VDR is finely regulated at multiple post-translational levels at baseline and in disease, which could potentially serve as targets for new treatments.

## Concluding remarks

Mineral metabolism hormones are tightly regulated at multiple levels – transcriptional, post-translational, secretion and interaction level. While there is significant understanding about genetic regulation, epigenetic regulation is not as thoroughly investigated. Many of the epigenetic studies are based on correlations and open the area for mechanistic studies and possible pharmacologic or genetic modifications. This could serve as novel therapeutics in mineral metabolism and beyond, e.g., cell cycle and energy metabolism modifications.

## Author contributions

IP and PS conceived the framework and main text of this review article. IP and PS wrote the draft and reviewed the manuscript. All authors contributed to the article and approved the submitted version.

## Funding

This article was funded by NIH Grant #1K08DK124568-01.

## Conflict of interest

The authors declare that the research was conducted in the absence of any commercial or financial relationships that could be construed as a potential conflict of interest.

## Publisher’s note

All claims expressed in this article are solely those of the authors and do not necessarily represent those of their affiliated organizations, or those of the publisher, the editors and the reviewers. Any product that may be evaluated in this article, or claim that may be made by its manufacturer, is not guaranteed or endorsed by the publisher.
